# Uterine Dysfunctions in Non‐Pregnant and Pregnant Small Animals

**DOI:** 10.1111/rda.70281

**Published:** 2026-09-02

**Authors:** Sandra Goericke‐Pesch

**Affiliations:** ^1^ Unit for Reproductive Medicine – Clinic for Small Animals University of Veterinary Medicine Hannover, Foundation Hannover Germany

**Keywords:** endometritis, pyometra, subinvolution of placental sites, uterine inertia

## Abstract

Various uterine dysfunctions in non‐pregnant and pregnant dogs and cats exist and can have a significant impact on the animal's health and welfare, as well as the offspring in case of pregnant and postpartum animals. This article aims to provide an overview, focusing on pyometra, endometritis, and uterine inertia, mainly in dogs.

## 1 Introduction

Various uterine dysfunctions in non‐pregnant and pregnant dogs and cats exist and can have a significant impact on the animal's health and welfare. In pregnant and postpartum animals, they additionally carry a significant risk for the offspring.

Pyometra is the most common uterine disease in non‐pregnant dogs and cats. Cystic endometrial hyperplasia (CEH), the cystic dilatation of the endometrial glands, is often considered a pre‐stage of pyometra. Nevertheless, CEH can also occur independently. Its frequency increases with increasing age (Moxon et al. 2016; Reusche et al. 2018), often concomitant with ovarian pathologies, such as follicular cysts or granulosa cell tumours. Because of CEH, but also independently sterile fluid might accumulate in the uterus causing mucometra, hydrometra, or haemometra. These pathologies are often associated with no or mild clinical signs, especially when infection is absent (Fransson et al. 2004; Hagman et al. 2006). A very specific condition is the pseudoplacentational endometrial hyperplasia (PEH) that is characterized by uterine lesions similar to placentation sites occurring during luteal phase due to unknown aetiology (Gifford et al. 2014). During ultrasound examination, this finding can be misinterpreted with pregnancy; however, no embryo is visible (Bracco et al. 2026). Endometritis, the inflammation of the uterine mucosa not extending beyond the stratum spongiosum, is another important dysfunction of the non‐pregnant uterus. Subclinical endometritis is likely the most underdiagnosed cause for sub‐ and infertility in dogs and cats (Fontbonne et al. 2020). Furthermore, uterine neoplasia is related to uterine dysfunction in non‐pregnant small animals.

Postpartum metritis (PPM) and subinvolution of placental sites (SIPS) are characteristic postpartum diseases. PPM typically occurs 1 week after parturition (Gonzales 2018; Lection et al. 2021), is infectious and can be life‐threatening due to septicaemia. SIPS, the prolonged sanguineous vaginal discharge post‐partum due to persisting and invading fetal cells in the placental sites, is usually non‐infectious and spontaneous recovery is common, despite various treatment options have been described: ecbolic drugs (oxytocin, prostaglandin (PG) F2α, ergot alkaloids), antibiotics and progestins (Sontas et al. 2011; Voorhorst et al. 2013). Beyond clinical findings and vaginal cytology (PPM: neutrophils; SIPS: trophoblast‐like cells), ultrasound is the diagnostic of choice (Bracco et al. 2026). Uterine prolapse is common during or right after parturition, especially in cats (Biddle and Macintire 2000; Davidson and Barber 2017), but has also been described in a non‐pregnant dog and cat (Greiling et al. 2023; Valentine et al. 2016).

In the following, pyometra, endometritis, and uterine inertia will be reviewed in more detail, focusing mainly on dogs.

## 2 Pyometra

Pyometra is the most common uterine disease in intact non‐pregnant dogs and cats. It is characterized by a pus accumulation in the uterus and can be life‐threatening. As several comprehensive, also recent reviews exist about pyometra (Hagman 2018, 2022; Xavier et al. 2023), this review only aims to summarize the key features.

Pyometra generally affects middle‐aged to older bitches and queens; however, a higher frequency is observed in bitches compared to cats. Despite pyometra in bitches being detectable at any time during the estrus cycle, progesterone (P4) plays a key role in the pathophysiology of the development of the disease. Clinical signs typically occur 6–10 weeks after heat in bitches and after ovulation and/or mating in queens. P4 stimulates growth and proliferation as well as secretion of the endometrial glands, cervical closure, suppression of myometrial contractions, and leucocyte inhibition (Hagman 2023). Besides hormonal factors, bacteria play an important role in the complex pathogenesis of pyometra. Although various bacteria can be involved and uterine dysbiosis was identified in affected bitches, most often *Escherichia (E.) coli* is found (Hagman 2023). Further characterization of the “pyometra *E. coli* strains” revealed a higher prevalence of phylogroup B2 in the uterus and gut compared to healthy animals (Mateus et al. 2013). Besides, specific virulence factors, e.g., the gene encoding type P fimbriae (papC), have been postulated to be involved in the pathogenesis (Krekeler et al. 2013), and the combination of virulence factors may determine the severity of disease (Lopes et al. 2021; Xavier et al. 2022). Certain strains with specific virulence factors may be more likely to cause canine pyometra by facilitating tissue colonization and even modifying the uterine environment favouring infection (Xavier et al. 2023). In cases of concomitant persistent bacteriuria, whole‐genome sequencing studies indicate that the same *E. coli* clone is causative for both. Camozzi et al. (2026) recently confirmed that the intestinal microbiota are a relevant source of infection in affected bitches, as *E. coli* isolates from the intestinal and reproductive tracts are genetically related. Investigating multi‐drug‐resistant *E. coli* derived from these cases confirms that specific international high‐risk clones exist (Sroithongkham et al. 2024). The observation that gut microbiota and diet seem to influence colonization by pyometra‐causing *E. coli* deserves further attention. Possibly, modulating the (gut) microbiota might be an additional option to reduce the risk of development (Xavier et al. 2022).

In both, dogs and cats, breed‐predispositions have been described with additional age‐differences in some dog breeds, i.e., specific breeds suffer from pyometra significantly earlier than the average of dogs and/or other breeds, respectively (Arendt et al. 2021; Egenvall et al. 2001; Hagman et al. 2009; Hagman et al. 2014; Jitpean et al. 2012; Kenney et al. 1987; Niskanen and Thrusfield 1998). This suggests possibly even breed‐specific genetic risk factors, such as in Golden Retrievers the region on chromosome 22, localized in the *ABCC4* gene, encoding a prostaglandin transporter (Arendt et al. 2021). Beyond those factors, concomitant presence of oestrogens and P4, either by exogenous hormone administration or co‐existence of corpora lutea and follicular cysts, is considered to be associated with an increased risk (Hagman et al. 2011; Ström Holst et al. 2001). CEH can predispose to pyometra, but can also occur independently (Schlafer and Gifford 2008).

Clinical signs are quite variable and only partly depend on the degree of uterine distension related to the disease. Whereas the diagnosis in open‐cervix pyometra is straightforward, it might be more challenging in closed‐cervix pyometra, lacking the characteristic putrid and/or hemorrhagic vaginal discharge. Severe illness and septicemia are more common in case of closed cervix, but the outcome does not differ (Jitpean et al. 2017). As all patients can rapidly deteriorate, seeking veterinary advice and intervention rapidly is strongly recommended. Surgical ovariohysterectomy remains the treatment of choice, especially in cases with severely disturbed general condition and/or concomitant disease (e.g., follicular cysts, CEH). Medical options, especially the use of antiprogestins with/without concomitant administration of prostaglandin (PG) F2 α may be an option in bitches with P4 above 2 ng/mL if intended for breeding. Concomitant treatment with antimicrobials is common, no matter if surgical or medical treatment options are chosen. To avoid antimicrobial resistance development and to ensure selection of the appropriate antimicrobial, bacterial culture is recommended. Bertero et al. (Bertero et al. 2024) suggested not to change the antibiotic in uncomplicated cases, even if the first choice was not right according to bacterial susceptibility testing. Ylhäinen et al. (Ylhäinen et al. 2023; Ylhäinen et al. 2025) even postulated that a single perioperative IV dose of sulfadoxine‐trimethoprim appears to be sufficient for preventing postoperative surgical site infections in uncomplicated pyometra cases as additional 5 days treatment of post operative sulfadiazine‐trimethoprim PO was not superior to a single perioperative treatment. New treatment options might also include biofilm disruptors as relevant *E. coli* strains produce biofilms in vitro and in vivo (Fiamengo et al. 2020).

## 3 Endometritis

Despite endometritis has been investigated in detail in various species, knowledge in dogs and especially cats is still limited: Similar to other species, acute endometritis is characterized by neutrophils in the endometrium, presence of hyperemia, vascular congestion, and/or stromal edema. In subacute endometritis, edema, polymorphonuclear, and mononuclear cells are found, whereas chronic endometritis reveals lymphocytes, plasma cells, and macrophages in the endometrium as well as interstitial fibrosis (Garcia Mitacek et al. 2017; Gifford et al. 2014; Praderio et al. 2019; Schlafer 2012). Endometritis is often hypothesized to be one of the most common causes for sub‐ and infertility in dogs and cats: Evaluating 399 canine uterine biopsies for reproductive health control or because of subfertility, 42.6% of cases suffered from endometritis, with 52.4% of these cases being considered chronic. CEH was the second most common diagnosis (33.3%) (Gifford et al. 2014), supporting also its role in canine sub‐ and infertility.

Exposure to semen and bacterial contamination from the vagina, after e.g., faecal contamination, insemination, parturition or abortion are possibly involved in the aetiology. The immunosuppressive mode of P4 action, reduced leucocyte migration and the absence of bacteria‐neutralizing uterine secretions (Dhaliwal et al. 2001) combined with the canine specific sustained P4 exposure after heat independent of mating might explain the relatively high incidences. Prolonged uterine fluid retention after mating visible during ultrasound examination indicates reduced uterine clearance and is a good marker for (subclinical) endometritis (Fontbonne et al. 2020; Freeman et al. 2013), especially with preexisting uterine conditions as CEH (England et al. 2021). The fluid was shown to reduce sperm attachment and fertilization rates. The direct diagnosis requires sampling from the uterus, either transcervically by endoscope (Arioni et al. 2025; Fontaine et al. 2009; Watts et al. 1998; Watts and Wright 1995) or by surgery (biopsy) (Mir et al. 2013), with the latter being considered golden standard. For identification of clinically “silent” endometritis, gene expression analysis of *leucocyte inhibitory factor, progesterone receptor (PGR*) and *Interleukin‐6* using transcervical lavage‐derived endometrial cells have been proposed as powerful potential molecular markers (Mitacek et al. 2023).

To prevent significant inflammatory changes after mating, prophylactic administration of trimethoprim‐sulfonamides in predisposed CEH bitches was suggested (England et al. 2012) similar to prophylactic use of NSAIDS to subdue inflammation (England et al. 2021; Roos‐Pichenot and Zakošek Pipan 2025). Despite treatment options are not evidence‐based, it seems unquestionable that early identification and suppression of the inflammation are key factors to prevent permanent infertility. Treatment with cyclooxygenase2‐inhibitors should be initiated only after ovulation, as this enzyme is crucial for ovulation in many species (Gaytan et al. 2006). A case report in the dog showed a temporary P4 drop following a single meloxicam injection in the periovulatory period, without, however, a long‐term effect on P4 and establishment of pregnancy (Dzieciol et al. 2022).

## 4 Uterine Inertia—A Specific Dysfunction During Parturition

Disturbances in the complex hormonal regulation of canine parturition can lead to dystocia and uterine inertia (UI), the failure of effective uterine contractions, is with over 70% of cases the most common cause (Darvelid and Linde‐Forsberg 1994; Davidson 2011). If myometrial contractions are too weak or not triggered at all, expulsion of the puppies fails. Uterine inertia can be further classified as primary or secondary, with differing definitions depending on the author (Davidson 2011). Generally, primary uterine inertia (PUI) is defined as the failure or complete absence of sufficient uterine contractions to expel normal sized puppies through a non‐obstructed birth canal. In complete PUI, second stage labor signs are lacking and thus no puppy can be delivered (Linde Forsberg 2010). In contrast, in partial or incomplete PUI second stage labor starts normally, but uterine contractions stop before the pup is expelled (Bennett 1974). Some authors also extend this definition to the cessation of contractions after the first pups have already been born, but before the entire litter has been expelled (Bennett 1974; Darvelid and Linde‐Forsberg 1994). Myometrial exhaustion caused by very large litter size is sometimes described as a reason for PUI or secondary uterine inertia (SUI) (Bennett 1974; Cornelius et al. 2019). SUI occurs when, following a physiological start of parturition, a prolonged phase of uterine contractions causes myometrial fatigue. SUI is usually the consequence of an obstruction of the birth canal (Gaudet 1985; Linde Forsberg and Persson 2007).

The underlying aetiology of PUI is considered multifactorial: predispositions of breed (Boxers, Labrador Retrievers, some Terrier breeds (Darvelid and Linde‐Forsberg 1994; Linde Forsberg and Persson 2007)), increased age and body weight, but also litter size (extremely small or large litters) (Linde Forsberg and Persson 2007; Münnich and Küchenmeister 2009). Failure of prepartum luteolysis was postulated causative (Bergstrom et al. 2010), but was not confirmed by other authors (Davidson 2011; Jungmann et al. 2022; Tamminen et al. 2019). P4 plays, however, without a doubt an important role: *Progesterone receptor* (*PGR*) gene expression was increased in interplacental tissues in PUI compared to obstructive dystocia (OD) (Jungmann et al. 2022) possibly pointing towards an increased responsiveness to any remaining P4 and prolonged quiescence state of the myometrium. Additionally, increased *PGR* expression was identified despite unchanged numbers of maternal decidual cells in placentas of PUI bitches compared to placentas of bitches during prepartum luteolysis (Steiner et al. 2025) supporting the hypothesis of disrupted P4‐PGR signalling. Besides, no clear relationship between oxytocin, vasopressin, estradiol, cortisol and PGF2α (measured as PGFM) and dystocia as well as UI, respectively, has been established by now (Bergstrom et al. 2006; Bergstrom et al. 2010; Hollinshead et al. 2010; Tamminen et al. 2019) and genes related to prostaglandin synthesis and pathways were not altered between PUI and OD (Rempel, Korber, et al. 2021; Rempel, Lillevang, et al. 2021). The role of the *oxytocin receptor* (*OXTR*) gene expression is controversial (Jungmann et al. 2022; Tamminen et al. 2019). Other factors possibly involved in the UI aetiology include systemic diseases of the bitch, alterations in calcium metabolism (hypo−/hypercalcemia) and nutritional imbalances (Linde Forsberg and Persson 2007; Münnich and Küchenmeister 2009). Despite the role of blood calcium concentrations is still unequivocal (Frehner et al. 2018; Hollinshead et al. 2010; Kraus and Schwab 1990), low blood calcium might contribute to the development of UI, especially in small breed dogs (Frehner et al. 2018). Defects or rather altered (gene) expressions, e.g., of the *myosin light chain kinase 4 (MLCK4*), the *myosin heavy chain 2* (*MHC2*) and the *protein kinase C (PKC)* have been described (Magnus et al. 2023), as well as alterations in cell–cell‐signalling (Rempel et al. 2022) – it remains, however, to be clarified, whether this is a cause or a consequence.

Diagnosis is made by clinical history, and possibly tocodynamometry to monitor uterine activity (Davidson 2011; Schröder 2008; Tamminen 2020). Independent of the dystocia cause, immediate recognition and therapeutical intervention are crucial, as UI presents a severe risk to fetal and maternal health and survival (Darvelid and Linde‐Forsberg 1994; Münnich and Küchenmeister 2009). As in case of uterine inertia, medical treatment with oxytocin, calcium and/or denaverine hydrochloride often fails; C‐section is often the treatment of choice.

## 5 Conclusion

Uterine dysfunctions in dogs and cats encompass a broad spectrum of disorders that differ in aetiology, clinical presentation, and consequences, yet all have the potential to compromise reproductive performance, maternal health, and offspring survival. While pyometra has been extensively investigated and significant progress has been made in understanding its pathogenesis and optimizing treatment strategies, substantial knowledge gaps remain regarding the pathophysiology, diagnosis, and evidence‐based management of conditions such as endometritis and primary uterine inertia, despite recent advances providing new insights into the mechanisms underlying these disorders and may facilitate the development of improved diagnostic tools and targeted therapeutic approaches. Future studies should focus on elucidating disease mechanisms, establishing standardized diagnostic criteria, and evaluating fertility‐preserving treatment strategies to improve reproductive health and reduce unnecessary antimicrobial use in companion animals.

## Author Contributions

Sandra Goericke‐Pesch: writing original draft preparation and finalization.

## Conflicts of Interest

The author declares no conflicts of interest.

## Data Availability

The data that support the findings of this study are available on request from the corresponding author. The data are not publicly available due to privacy or ethical restrictions.
